# Gender Differences: Examination of the 12-Item Bem Sex Role Inventory (BSRI-12) in an Older Brazilian Population

**DOI:** 10.1371/journal.pone.0076356

**Published:** 2013-10-02

**Authors:** Lisa F. Carver, Afshin Vafaei, Ricardo Guerra, Aline Freire, Susan P. Phillips

**Affiliations:** 1 School of Rehabilitation Therapy, Queen’s University, Kingston, Canada; 2 Department of Community Health & Epidemiology, Queen’s University, Kingston, Canada; 3 Departments of Family Medicine and Community Health & Epidemiology, Queen’s University, Kingston, Canada; 4 Department of Physiotherapy, Federal University of Rio Grande do Norte, Natal, Brazil; Johns Hopkins Bloomberg School of Public Health, United States of America

## Abstract

**Objectives:**

Although gender is often acknowledged as a determinant of health, measuring its components, other than biological sex, is uncommon. The Bem Sex Role Inventory (BSRI) quantifies self-attribution of traits, indicative of gender roles. The BSRI has been used with participants across cultures and countries, but rarely in an older population in Brazil, as we have done in this study. Our primary objective was to determine whether the BSRI-12 can be used to explore gender in an older Brazilian population.

**Methods:**

The BSRI was completed by volunteer participants, all community dwelling adults aged 65+ living in Natal, Brazil. Exploratory factor analysis was performed, followed by a varimax rotation (orthogonal solution) for iteration to examine the underlying gender roles of feminine, masculine, androgynous and undifferentiated, and to validate the BSRI in older adults in Brazil.

**Results:**

The 278 participants, (80 men, 198 women) were 65–99 years old (average 73.6 for men, 74.7 for women). Age difference between sexes was not significant (p = 0.22). A 12 item version of the BSRI (BSRI-12) previously validated among Spanish seniors was used and showed validity with 5 BSRI-12 items (Cronbach=0.66) loading as feminine, 6 items (Cronbach=0.51) loading onto masculine roles and neither overlapping with the category of biological sex of respondent.

**Conclusions:**

Although the BSRI-12 appears to be a valid indicator of gender among elderly Brazilians, the gender role status identified with the BSRI-12 was not correlated with being male or female.

## Introduction

Many researchers use the terms sex and gender interchangeably. However, sex is defined physiologically whereas gender is a cultural construct that includes social and psychological factors [1]. Gender is linked to roles and behaviors expected of men and women in a particular culture at a specific time, and may be influenced by education and socioeconomic status. Sex and gender roles are “intertwined; they influence each other in a network of constant reciprocal changing processes” (p. 72) [2], however, it is possible to measure them separately. This research examines the ability of a short version of the Bem Sex Role Inventory (BSRI) to assess gender role in a sample of older Brazilian adults.

### Defining Gender Roles

Gender is an understudied area in health research, particularly among older adults. When it is considered, the most commonly used and repeatedly validated measure of gender roles is the Bem Sex Role Inventory (BSRI) developed 4 decades ago [3]. Sandra Bem categorized instrumental traits including: taking the lead, being aggressive, competitive, dominant, self-reliant, and athletic as masculine; while feminine role characteristics were considered expressive and included compassion, affection, sympathy, warmth, and being yielding.

Bem was the first to conceptualize gender roles as something other than exclusively masculine and feminine [3–5], defining a third role, androgyny, that combined both masculine and feminine traits and a fourth category, undifferentiated, describing people whose scores on both masculine and feminine traits were low [6]. She hypothesized that “many individuals might be "androgynous"; that is, they might be both masculine and feminine, both assertive and yielding, both instrumental and expressive—depending on the situational appropriateness of these various behaviors” (p. 155) [3]. Androgynous men and women were postulated to be adaptive and therefore more likely to have better mental health and higher competence [7]. Some dispute the androgyny model [8,9] while others assert that androgyny exists, but reflects a developmental change [10].

The original BSRI included 60 dichotomous items divided into 3 subscales (Masculinity, Femininity, and Neutral) of 20 items each [3]. A personality characteristic was categorized as feminine if it was independently judged, using a 7 point scale, by both females and males to be significantly more desirable for women than for men [11] and vice versa for masculine characteristics. Bem’s early studies [3] found alpha coefficients for masculinity of .86 and between .80 to .82 for femininity. Kamas and Preston [12] recently examined the BSRI with 310 college students determining that the scale remains valid.

Shorter versions of the BSRI are common. In 1981, Bem used factor loading to develop a 30 item scale, with 10 items per subscale [11], validated independently by others [13,14]. Following further confirmatory factor analysis Campbell, Gillaspy and Thompson [13] recommended using the shortened BSRI in future research due to its high reliability (α_M_ = .82, α_F_ = .89). A 12 item Spanish version of the BSRI (BSRI-12) was validated by Mateo and Fernandez [15] and included the items: gentle, sympathetic, leadership abilities, acts as a leader, dominant, tender, warm, affectionate, strong personality, defend own beliefs, sensitive to other’s need, and makes decision easily. This version demonstrated strong psychometric properties, in some cases better than the original 60 item BSRI [13,15,16].

**Figure 1:**
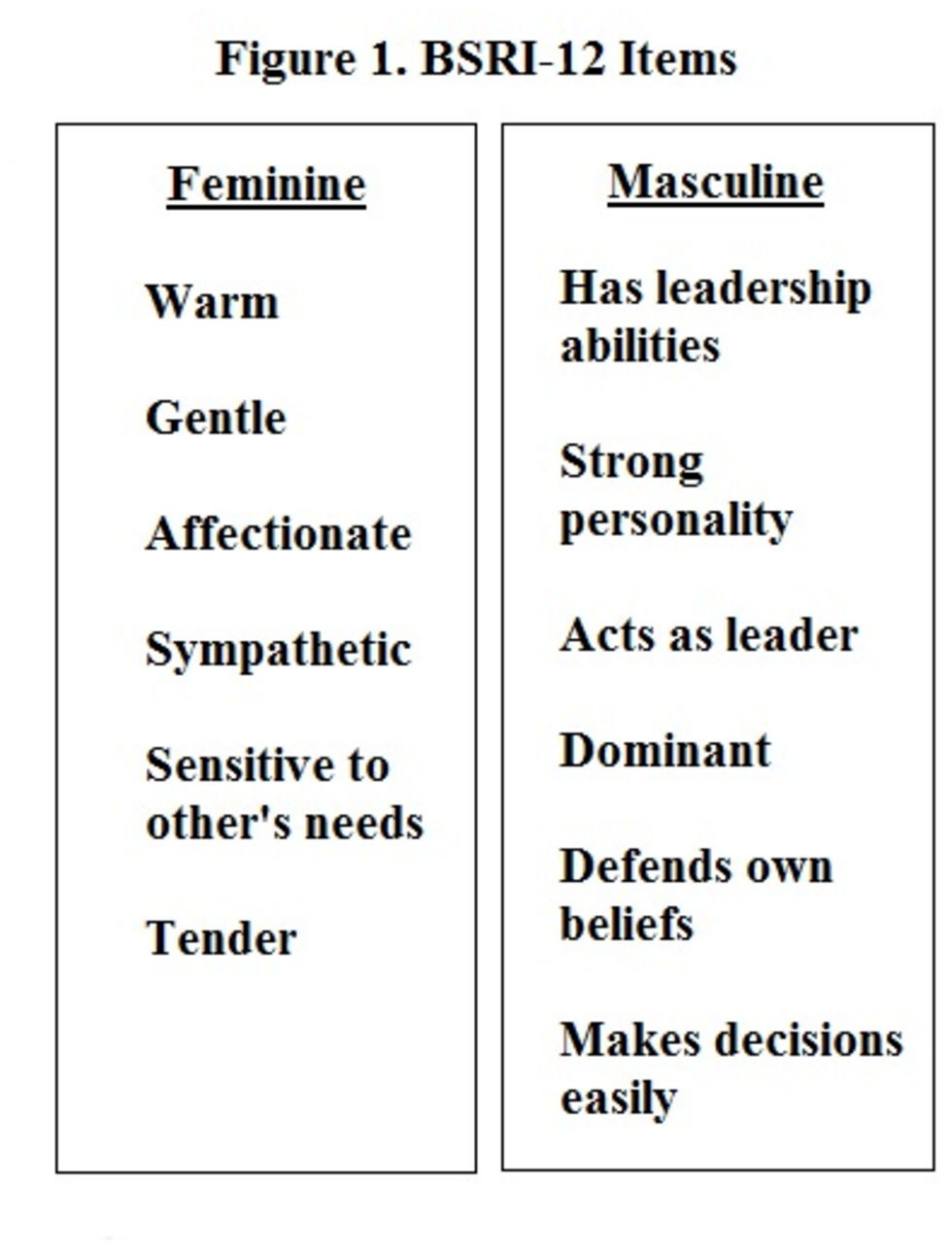
BSRI-12 Items.

Mateo and Fernandez [15] derived the 12 item BSRI (BSRI-12) from the original 60 item BSRI using a sample of Spanish university students. Students were told to evaluate the extent to which each item applied to him/her. A 7-point Likert scale was used: 1 signifying that the item was not applicable and 7 indicating the item totally applied. The BSRI-12 was translated into Spanish by one of the authors and then back-translated into English by two bilingual researchers, one of them was a U.S. citizen whose first language was English, the other was born and raised in Spain. Mateo and Fernandez [15] analyses resulted in a 12 item scale, shown in Figure 1, with six masculine/instrumental (M/I) items (defends own beliefs, strong personality, has leadership abilities, makes decisions easily, dominant, acts as a leader) and six feminine/expressive (F/E) items (affectionate, sympathetic, sensitive to needs of others, warm, tender, gentle).

Fernandez and Coello [16] used the BSRI-12 more recently, with students ranging in age from 12 to 15 years. They found reasonable reliability, using Cronbach’s alpha, on the anticipated Feminine/Expressive (F/E) and Masculine/Instrumental (M/I) scales (F/E scale α = .77; M/I scale α = .73) indicating internal consistency. They performed factor analysis using principal axis exploratory factor analysis (EFA) with oblimin rotation and δ = 0, revealed three factors explaining 58.11% of the total variance. Fernandez and Coello [16] described these factors as F1 (F/E) which loaded feminine/expressive items exclusively, explained 25.9% of total variance; F2 (M/I) loading exclusively masculine items and explaining 23.68% of the variance; and F3 (with both M/I items and F/E items) and accounting for 8.52% of variance. They also performed a principal components analysis which resulted in the same three factors, which were not significantly intercorrelated.

### Gender Role Classification

Methods used to classify respondents’ scores into gender role vary and can impact the gender role attributed to a respondent [17,18]. Generally classification is done using participants’ scores on the masculine and feminine scales. Common methods for classifying scores on the BSRI into gender roles are to split the sample using either the medians from Bem’s original normative samples [18], the theoretical mean of the scale [16] or the sample medians [17–19].

Whether dividing the scores by theoretical mean or median (sample or normative) the technique is the same. For example, using the median split, Lenney [18] suggested that subjects should be classified as masculine (M) “if their M score is above the Median and their F score is below the F median; as feminine if the F score is above the F median, and their M score is below the M median; as androgynous if both their scores are above the respective scale medians; and as undifferentiated (or indeterminate) if both their scores are below the respective scale medians” (p. 579). Medians for the masculine and feminine scales are established using scores from the whole sample; however, Lenney [18] points out that some researchers use medians established in Bem’s normative studies. Caution must be used in considering the generalizability of the results of these methods. As Sedney [17] points out, using different sample medians to divide people into gender role classifications can result in “a subject who scored 5.00 on both the BSRI F and M scales might be called Androgynous at Stanford, Feminine at Harvard, Masculine at Michigan, and Undifferentiated at a liberal arts college in Rhode Island” (p. 219).

### International use of BSRI

The full 60 item and the short versions of the BSRI appear to be valid across geography and culture. The BSRI has been used, for example, in Zimbabwe [20], Japan, and China [21] with good validity and reliability when a few items were removed to improve cultural fit. Using a short version of only instrumental items in a thirteen nation study with 6,543 participants, mean age 38.5 years old, Arrindell, van Well, Kolk, Barelds, Oei et al. [22] demonstrated internal consistency (Cronbach α = .83 averaged across the thirteen countries) and a strong correlation (r = .57, p < 0.05) with the Masculine Gender Role Stress (MGRS) scale. Research using both Spanish and English versions of the BSRI among middle aged women (mean age 44 years) has demonstrated reliability of the masculine (α = .78 in English and .76 in Spanish) and feminine (α = .88 in English and .86 in Spanish) scales [23]. Although the Bem Sex Role Inventory has some history of reliability and validity in Brazil [24–26], to the best of our knowledge neither the full nor the 12 item BSRI has been evaluated among older Brazilians.

### Gender and Aging

Gender is often overlooked in studies of aging populations. When it is considered, the BSRI appears to be the measure of choice [19,27]. For both men and women, gender identity is less rigid among older individuals [28,29]. In the few studies that looked at gender roles within aging populations, findings suggest that gender roles and biological sex may not be related. Twenge [30] found that with aging, women’s masculinity, but not their androgyny, scores increase. Huyck [31] also suggested that women embrace instrumental traits as they age. For both men and women, gender identity is less rigid among older individuals [28,29]. Finding that BSRI scores were not clearly masculine, feminine or androgynous among seniors (mean age of 70.6) Windle and Sinnott [27] hypothesized that this reflected complex gender roles rather than a dichotomous construct.

Gale-Ross et al. [19] examined gender role, life satisfaction and wellness in older Canadian women. Among their sample of older adults, 40% (n = 18) of male participants were classified by median split as masculine (instrumental), as compared to 11% (n = 6) of female participants. Thirty-six percent (n = 20) of women were classified as feminine (expressive) as compared to 16% of males (n = 7). Interestingly, 24% of males (n = 11) and 26% of females (n = 14) were classified as androgynous. More women (27%, n = 15) than men (20%, n = 9) were classified as undifferentiated. The variation between men and women in the gender classifications was not statistically significant. Gale-Ross et al. [19] concluded that their small sample size and lack of diversity may have played a role in their gender role results. They did find that androgyny was significantly associated with life satisfaction and general wellbeing.

Given the limited research on gender and older adults our primary objective was to determine whether the BSRI-12 can be used to explore gender in an older Brazilian population. Consistent with postulates underlying the BSRI, we hypothesized that men would be disproportionately represented in the masculine (instrumental) category while women would fall in the feminine (expressive) category on the BSRI.

## Methods

### Participants

Three hundred participants were randomly sampled from among community dwelling adults (older than 64 years) living in Natal, the capital of the province Rio Grande do Norte, Brazil. Two hundred and seventy-eight individuals (93%, 80 men, 198 women) agreed to participate in the study. Participants’ ages ranged from 65 to 99 with an average of 74.4 years. Marital status is given in Table 1. Fewer than half the participants (45%) were married; almost one third (32%) were widowed, the remainder were single or divorced. Men were, on average, younger than women (73.6 vs. 74.7), however the difference was not significant (*t*-test p value=0.22). Ninety-three percent of participants had fewer than six years of education (Table 2). Only 42% were satisfied with their level of income. Despite variations in socio-demographic factors within the groupings by sex, differences across sexes were not significant (Table 2).

**Table 1:** Marital status of participants.

	Married (%)	Single (%)	Widowed (%)	Divorced (%)	Total (%)
Men	38 (48)	12 (15)	26 (32)	4 (5)	80 (100)
Women	86 (43)	34 (17)	36 (32)	15 (8)	198 (100)

**Table 2:** Description of the population, none of differences between males and females were significant (*t*-test for age and Chi-Square tests for categorical variables).

Variable	Men (n=80)	Women (n=198)	Total (n=278)
Age (mean, SD)	73.6 (6.7)	74.7 (6.9)	74.4 (6.9)
Married (%)	38 (48)	86 (43)	124 (45)
Education			
≤ 5 years	65 (81.25)	186 (94)	258 (93)
6 years	5 (6.25)	8 (4)	13 (5)
7 years	9 (11.25)	4 (2)	6 (2)
9 years	1 (1.25)	0(0)	1 (.36)
Income Adequate	39 (49)	79 (40)	118 (42)

### Ethics Statement

Written informed consent was granted by all participants. This project was approved by the Committee on Ethics and Research of the University Hospital Onofre Lopes.

### Instruments

#### Socio-demographic factors

Information including age, sex, and marital status was collected for each participant. In addition, socioeconomic status (SES) of participants was assessed via level of education and satisfaction with income.

### Gender roles

This study used the BSRI-12 (English items listed in Figure 1.) translated from Spanish [15] to Portuguese by two Brazilian translators familiar with the concept of gender roles. Both Brazilian and Canadian researchers performed back translation [24]. As described above, BSRI-12 measures participants’ instrumental (masculine) and expressive (feminine) traits and thereby, gender roles. A 7-point Likert scale was used: 1 represented ‘not applicable’ and 7 represented ‘totally applicable.’ In the Spanish version of the BSRI-12 Mateo and Fernandez [15] found the coefficient of internal consistency [32] was .83 to .94.

### Data Analysis

#### Descriptive

To obtain a general overview of the population under study, descriptive statistics for each category of SES were calculated. Results are reported for the total population, as well as stratified by sex.

#### Factor analysis

The BSRI-12 was subjected to an exploratory factor analysis to validate it as a measure of gender roles in this older population. The principal axis method was used to extract underlying factors, followed by a varimax rotation (orthogonal solution). The reason for choosing orthogonal solution over oblique solution was that gender constructs extracted in initial analyses were not correlated [33]. Two standard methods were employed to determine the number of factors to retain: Kaiser Criterion, which retains factors with Eigenvalues greater than 1 [34], and Cattell’s [35] scree test, which involves an examination of a plot of the Eigenvalues for breaks or discontinuities.

In interpreting the rotated factor pattern, an item was said to load onto a given factor if the factor loading was greater than or equal to 0.25. Items which load on two factors similarly will not provide a meaningful interpretation [33]. To establish *a priori* criteria, if the differences between loading on two or more factors were smaller than 0.10, the item was excluded from analysis [33]. To assess the degree to which items included in each factor measured the same underlying concept (internal reliability, consistency), Cronbach’s Coefficient Alpha was computed for each of the retained factors [36].

#### Classification into Gender Roles

**Figure 2:**
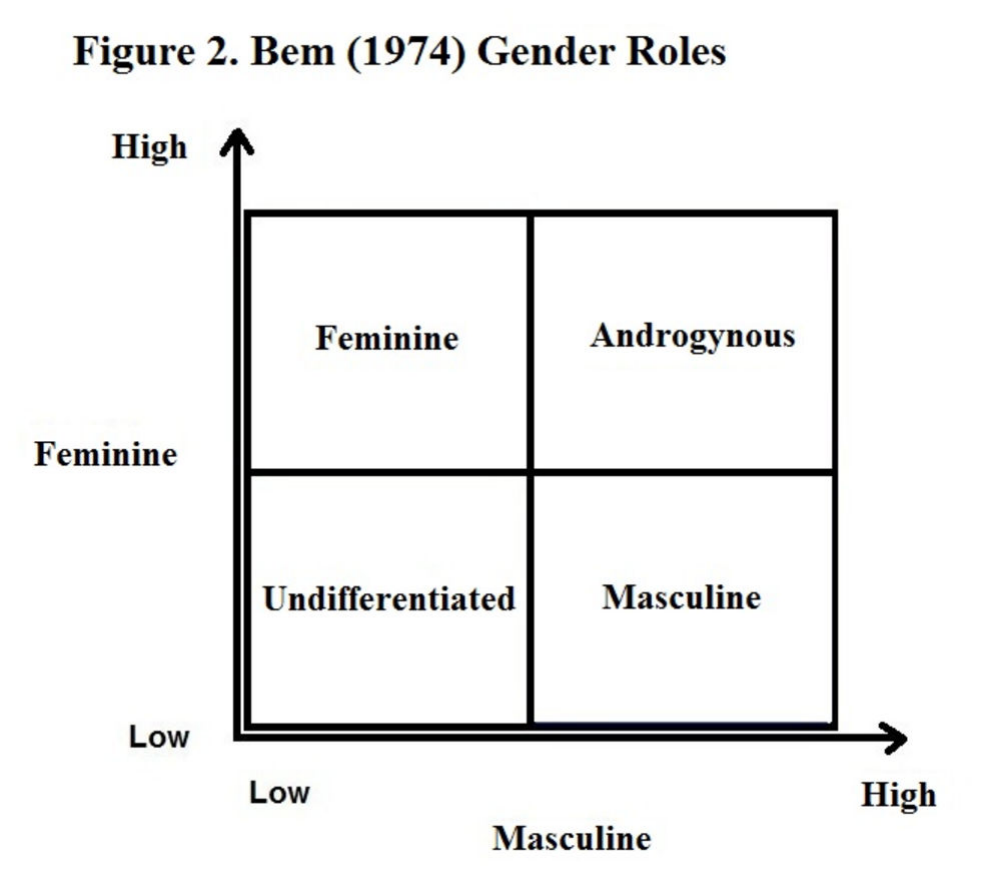
Bem (1974) Gender Roles.

In the literature there are multiple methods for classifying people into gender roles. The most common method uses the median split. This method was used in a recent study of an elderly population [19] and avoids methodological issues that occur when other approaches are used [37]. Therefore the median split method was used to classify the gender roles of these participants. First the median for the whole sample was established for both the masculine and the feminine scales. Then individual scores for each participant on the femininity scale and the masculinity scale were calculated and compared to the median. Scores that fell at the median were classified as “high” rather than “low” scores. If the individual’s mean score was below the median on both the feminine and masculine scales, he/she was classified as undifferentiated. If the individual’s mean scores on both the masculine and feminine scales were equal to or above the median that individual was classified as androgynous. Those people who were equal to or higher than the median on the feminine scale and lower on the masculine scale were classified as feminine. Finally, those who were equal to or higher than the median on the masculine scale and lower on the feminine scale were classified as masculine (see Figure 2).

## Results

Prior to performing factor analysis the Kaiser-Meyer-Olkin (KMO) measure of sampling adequacy (KMO = .71) was used to ascertain the amount of item-variance shared among variables in the input matrix. Given that the KMO was greater than .70, the input matrix was deemed adequate in size [38]. The Bartlett’s Test of Sphericity (χ^2^ = 357.8, df = 66, p < .0001) was statistically significant, demonstrating that the input correlation matrix was not equal to zero and therefore was suitable for factor analysis.

The exploratory factor analysis with varimax rotation resulted in factor loadings presented in Table 3. Results suggested retention of two factors based on our previously defined criteria. Factor 1 loaded feminine/expressive (F/E) items and accounted for 22% of the total variance. The second factor loaded masculine/instrumental (M/I) items and explained 12% of the variance. The inter-factor correlation (0.39) was below .50, the cut-off point recommended by Gorsuch [39] so factor analysis was deemed acceptable.

**Table 3:** Factor Analysis pattern matrix (12 items).

Factor 1 (Feminine)	Loading	Factor 2 (Masculine)	Loading
Warm	0.62	Has leadership abilities	0.50
Gentle	0.48	Strong personality	0.49
Affectionate	0.77	Acts as leader	0.40
Sympathetic	0.39	Dominant	0.28
Sensitive to other’s needs	0.36	Defends own beliefs	0.28
Tender*	-0.06	Makes decisions easily	0.28
Cronbach’s alpha	0.66	Cronbach’s alpha	0.51

* Was not included in reliability calculation due to very small loading

The five items loading onto factor 1 (F/E) were: warm (0.62); gentle (0.48); affectionate (0.77); sympathetic (0.39); sensitive to other’s needs (0.36). All were feminine items in the original inventory and showed reasonable internal consistency (Cronbach’s alpha=0.66). All six items loading onto factor 2 (M/I) were masculine items: leadership abilities (0.50); strong personality (0.49); acts as a leader (0.40); dominant (0.28); and defends own belief (0.28). The Cronbach’s alpha (α=0.51) suggested weak internal consistency among these items. One item, ‘tender,’ did not load onto either factor and was, therefore, excluded from further analysis.

### Gender role groups based on the Bem androgyny model

The Bem androgyny model [3] was the conceptual base for constructing four gender role groups. Using a median split: among men, 18% (n=14) were categorized as masculine, 32% (n=26) were androgynous, that is, high in both masculine and feminine items, 16% (n=13) were categorized as feminine and 34% (n=27) were undifferentiated. For women, 20% (n=40) were classified as feminine, 19% (n=37) were masculine, 34% (n=67) were androgynous and 26% (n=52) were undifferentiated (Table 4). Most importantly, perceived gender roles did not differ significantly by sex (t(274) = -.737, p < .46), suggesting that biological sex is a different entity than gender.

**Table 4:** Gender roles across biological sexes.

Gender role group	Male sex (%)	Female sex (%)
Undifferentiated	27 (34)	52 (26)
Masculine	14 (18)	37 (19)
Feminine	13 (16)	40 (20)
Androgynous	26 (32)	67 (34)
Missing		2 (1)
Total	80 (100)	198 (100)

There was no significant difference between males scores and female scores (t(274) = -.737, p < .46).

In summary, our factor analysis showed that, in this population, the BSRI-12 differentiated two factors, corresponding to feminine and masculine scales. Contrary to our predictions, gender role classification did not reflect biological sex. In fact a higher percentage of both males and females were classified as either androgynous or undifferentiated than those classified in traditional gender roles of masculine and feminine. Furthermore, the lack of association between biological sex and gender roles indicates that sex and gender roles are different entities in this population. 

## Discussion

There is growing evidence that gender roles have an effect on health that is independent of biological sex itself, with masculinity likely conferring greater risks of illness for both men and women [40–44]. The most utilized measure of gender roles is the BSRI, developed almost 4 decades ago and validated repeatedly either as a 60 item inventory or an abbreviated form. Asking about, and including measures of gender roles among an older, non-English speaking population is surprisingly rare in health outcomes research. Perhaps this is because translating the concepts in the inventory is not simple and straightforward, but it may also speak to assumptions that seniors are either unaffected by gender roles or that they all ascribe to the same roles. If research is to acknowledge and account for gender as a social determinant of health then measuring gender roles is a key component of such research. Gender, however, is not fixed or static but varies across time and place making repeated validation of measures such as the BSRI necessary as characteristics of any population studied vary [22].

It would appear that the gender role differentiation power of BSRI-12 was not as strong statistically in our research as in a number of earlier studies. There are several possible explanations for this. Although the BSRI-12 could separate gender roles among this group, Cronbach’s alphas were below the traditionally acceptable limit of .70, perhaps because, as Streiner [45] explains, measures with fewer items result in reduced alpha’s. We also found different factor loadings than in previous studies using the BSRI-12 [16]. Despite such variations in loadings and Cronbach’s alphas, other researchers’ results consistently list items on the BSRI-12 among the items on their first two factors [13]. It may be that over the decades and with changes in gender roles the BSRI will require modification to better reflect social expectations of men and women. This is in keeping with the fluid and contextual nature of gender, itself.

Our original hypothesis was that a greater proportion of males would be classified in the masculine/instrumental category and a larger proportion of females would be classified as feminine/expressive. This was not the case. Similar to other researchers studying older adults [19], we found little overlap between biological sex and gender roles; that is, men were not significantly more likely to be masculine than were women. In fact, a smaller percentage of males and females fell into the traditional gender roles of masculine and feminine than into the androgynous and undifferentiated categories. In this sample of older Brazilians, unlike Twenge’s [30] finding that masculinity scores, rather than androgyny scores, increase in aging women, we found that more men (32%) and women (34%) were androgynous than masculine (men 18%; women 19%).

The lack of differentiation according to physiological sex between gender roles may have been due to the translation of the instrument, classification method or the low education and socioeconomic status of our participants. Perhaps the BSRI-12 does not describe masculinity and femininity in ways that fit the context of our study population. On the other hand, and in keeping with other research [19,26] this lack of congruency between biological sex and gender roles may indicate that each is an independent contributor to who a person is and, potentially, to well-being; and that measuring both constructs will add explanatory value in social determinants research. Despite frequent assumptions that the elderly are more traditional than younger people, the lack of overlap between sex and gender roles in this older population from a relatively traditional culture suggests that with aging, gender roles may actually become less stereotypic and rigid, even in a more traditional and less egalitarian society.

## Conclusion

To deepen the meaning of biological sex and address how or whether aspects of gender are determinants of health requires including measures of gender equalities, constraints and expectations in quantitative research. The BSRI is one such measure that, despite its age, remains valid, and adds meaning not captured by the simple dichotomous classification of sex. It does require revalidation when used with new study populations.

Among a relatively socioeconomically deprived population of Brazilian seniors the BSRI-12 may have measured aspects of gender not encompassed by the construct of sex. Research on education, SES and gender may reveal the extent to which socioeconomic deprivation impacts gender. Using only sex in research automatically dichotomizes a population. Including the BSRI-12 as a measure of gender roles among this group of older Brazilian adults suggests convergence rather than division between men and women. And, given that fatigue is a common issue for older adults participating in research studies [46], using less time-consuming instruments, such as the BSRI-12, is an important strategy. The BSRI-12 appears to have meaning across contexts and age groups, and to be a valid measure of one aspect of gender among this older, low socioeconomic status, Brazilian population.
